# A Zero-Dimensional Zn(II)-Based Organic–Inorganic Hybrid Metal Halide with Blue-Green Emission for White Light-Emitting Diode Application

**DOI:** 10.3390/molecules31122082

**Published:** 2026-06-13

**Authors:** Hua-Peng Liu, Yu-Chen Wang, Zhen-Chao Hu, Yuan-Chun He

**Affiliations:** 1 Key Laboratory for Medical Functional Nanomaterials, College of Medical Engineering, Jining Medical University, Jining 272067, China; 2 Key Laboratory of Catalytic Conversion and Clean Energy in Universities of Shandong Province, School of Chemistry and Chemical Engineering, Qufu Normal University, Qufu 273165, China

**Keywords:** OIMHs, luminescent material, Zn(II), WLED

## Abstract

Organic–inorganic hybrid metal halides (OIMHs), especially zero-dimensional (0D) ones, have been recognized as an excellent class of luminescent materials due to their structural diversity and tunable emission properties. In this work, using the environmentally friendly Zn(II) ion as the central metal and 1,4,7,10-tetraazacyclododecane (Cyclen) as the organic component, we successfully synthesized a novel OIMH, (H_3_Cyclen)(ZnBr_4_)·Br·H_2_O. Single-crystal X-ray diffraction analysis reveals that (H_3_Cyclen)(ZnBr_4_)·Br·H_2_O possesses a 0D structure, in which the [ZnBr_4_]^2−^ tetrahedra are uniformly separated by the organic amine cations. This structural feature is expected to enhance the material’s stability and optimize its optoelectronic properties. Under UV lamp irradiation, (H_3_Cyclen)(ZnBr_4_)·Br·H_2_O emits bright blue-green light. Therefore, we systematically investigated its luminescence properties. The emission mechanism was further elucidated using UV–vis absorption spectroscopy and DFT calculations. Finally, (H_3_Cyclen)(ZnBr_4_)·Br·H_2_O was employed as a luminescent material to fabricate a white light-emitting diode (WLED), demonstrating its potential as an excellent phosphor material.

## 1. Introduction

With the rapid advancement of optoelectronic technology, luminescent materials have shown broad application prospects in fields such as solid-state lighting, bioimaging, information anti-counterfeiting, and photodetection [1,2,3]. Although traditional rare-earth-doped luminescent materials offer high efficiency and good stability [4,5,6], their high preparation cost, resource scarcity, and limited tunability of emission wavelengths restrict their large-scale applications. In recent years, researchers have focused on developing novel high-performance luminescent materials, particularly Pb(II)-free and environmentally friendly candidates with tunable emission, high quantum efficiency, and good processability [7,8,9]. Organic–inorganic hybrid metal halides (OIMHs) have emerged as a frontier in luminescent materials research due to their structural diversity and tunable photophysical properties [10,11,12,13].

Among the various OIMHs, zero-dimensional (0D) structures have attracted particular attention owing to their unique “host-guest” configuration. In 0D OIMHs, the inorganic metal halide polyhedra are effectively isolated by organic cations, forming isolated emission centers that significantly reduce non-radiative transitions, thereby enabling broadband emission, large Stokes shifts, and high photoluminescence quantum efficiency. Zn(II)-based hybrid halides, in particular, are considered ideal candidates for novel luminescent materials due to their non-toxicity, earth abundance, and tunable photophysical properties [14,15,16,17,18,19,20,21]. By adjusting the structure of the organic cations, the type of halogens, and the coordination environment of the metal center, the emission color and efficiency can be effectively modulated to meet the requirements of various applications. Therefore, the development of novel Zn(II)-based 0D OIMHs is significant at present. However, most studies focus on small organic amines (e.g., tetraethylammonium, piperazine) as countercations. Cyclen (1,4,7,10-tetraazacyclododecane) is a macrocyclic tetraamine that can be protonated to form a bulky, multiply charged cation. Its large size and hydrogen-bonding ability make it an excellent organic spacer to isolate inorganic unit, promoting a zero-dimensional structure that favors self-trapped exciton emission. We therefore selected Cyclen as a representative bulky cation to construct the new Zn(II)-based 0D OIMH.

In this work, a novel Zn(II)-based 0D OIMH, (H_3_Cyclen)(ZnBr_4_)·Br·H_2_O was successfully synthesized. Single-crystal X-ray diffraction analysis shows that the [ZnBr_4_]^2−^ tetrahedra are effectively isolated by protonated H_3_Cyclen^3+^ cations. Furthermore, (H_3_Cyclen)(ZnBr_4_)·Br·H_2_O were characterized by powder X-ray diffraction, infrared spectroscopy, and thermogravimetric analysis. The solid-state photoluminescence properties were systematically investigated and further analyzed using density functional theory (DFT) calculations. Moreover, the weak interactions within the crystal were revealed by Hirshfeld surface (HS) analysis. Finally, the material was successfully employed to fabricate white light-emitting diodes (WLEDs) by mixing with commercial red and green phosphors.

## 2. Results and Discussion

### 2.1. Crystal Structure Description

**Figure 1:**
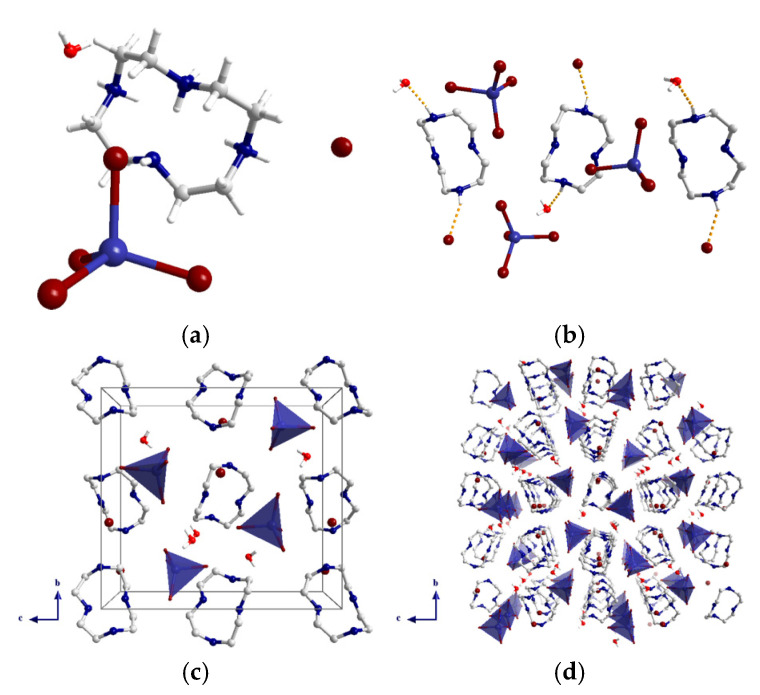
(**a**) Asymmetric unit diagram of (H_3_Cyclen)(ZnBr_4_)·Br·H_2_O. (**b**) Hydrogen bonds in the crystal lattice. Orange dashed lines represent N–H···O and N–H···Br bonds. (**c**) Unit cell packing diagram of the compound viewed along the a-axis. (**d**) Crystal packing structure of the compound viewed along the a-axis.

Analysis of the single-crystal X-ray diffraction data reveals that the compound (H_3_Cyclen)(ZnBr_4_)·Br·H_2_O crystallizes in the orthorhombic system with the space group *P*2_1_2_1_2_1_. The unit cell parameters are *a* = 7.682 Å, *b* = 15.184 Å, *c* = 16.576 Å, *α* = 90°, *β* = 90°, *γ* = 90°, *V* = 1933 Å^3^, *Z* = 4 (Table 1). Figure 1a,b show the asymmetric unit and hydrogen bonds of the compound. Each asymmetric unit contains one organic cation H_3_Cyclen^3+^, one [ZnBr_4_]^2−^ tetrahedron, one free water molecule, and one Br^−^ ion. The crystal structure contains two types of hydrogen bonds (N–H···Br and N–H···O), which connect the free Br^−^ ions and free water molecules to the organic Cyclen^3+^ cations, respectively. As shown in Figure 1c,d, the compound features a typical zero-dimensional structure, where the inorganic [ZnBr_4_]^2−^ units are uniformly separated by the organic H_3_Cyclen^3+^ cations.

**Figure 2:**
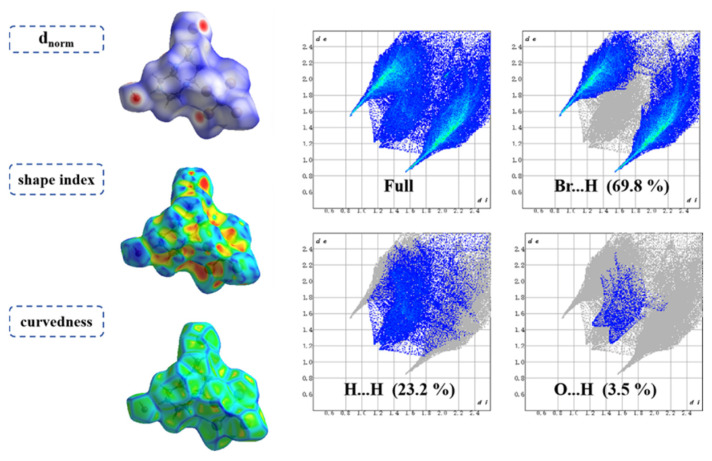
The d_norm_, shape index, and curvedness mapped Hirshfeld surfaces as well as the 2D fingerprint plots.

To analyze the intermolecular interactions within the compound, the Hirshfeld surface and the corresponding two-dimensional fingerprint plots were calculated and analyzed [22,23,24]. Figure 2 shows the Hirshfeld surface of (H_3_Cyclen)(ZnBr_4_)·Br·H_2_O mapped with shape index and curvedness, along with the 2D fingerprint plots. The fingerprint plots are symmetric, indicating a relatively uniform distribution of intermolecular contacts. The fingerprint plots reveal that Br···H contacts contribute the most (69.8%), followed by H···H (23.2%) and O···H (3.5%). On the d_norm_ surface, red spots appear around the free Br^−^ ions and water molecules, corresponding to the two types of hydrogen bonds in the structure: N–H···O and N–H···Br. In the 2D fingerprint plots, two sharp spikes are observed in the Br···H contact contribution, typically indicating strong interactions, which correspond to the N–H···Br hydrogen bonds in the crystal structure. In contrast, the H···H contact contribution appears as a broad peak, suggesting that the H···H interactions in the crystal structure are primarily van der Waals forces. Based on the structural and Hirshfeld surface analyses described above, the extension of the overall structure is achieved through weak interactions. This indicates that weak interactions play a crucial role in the formation of the structure. Moreover, the weak interactions between the inorganic and organic components separate the inorganic units from each other, a structural feature that is often beneficial to the photophysical properties of OIMHs.

### 2.2. Powder X-Ray Diffraction, Infrared Spectra and Thermogravimetric Analyses

Powder X-ray diffraction (PXRD) data were collected (Figure S1), and the measured patterns were compared with the simulated patterns derived from the refined single-crystal data. The excellent agreement in peak positions confirms that the as-synthesized compound is phase-pure and verifies the high accuracy of the single-crystal data.

The infrared spectrum was measured (Figure S2). For (H_3_Cyclen)(ZnBr_4_)·Br·H_2_O, the broad peak near 3400 cm^−1^ is attributed to the O–H of H_2_O and the N–H stretching vibration of the Cyclen ring. The multiple peaks in the range of 3000–2850 cm^−1^ arise from the C–H stretching vibrations of the methylene groups on the ring. The peaks in the range of 1300–1000 cm^−1^ are attributed to the C–N stretching vibrations, and the peaks in the range of 1000–800 cm^−1^ correspond to the skeletal vibrations of the macrocyclic ring.

To further investigate the thermal stability of the compounds, thermogravimetric analysis (TGA) was performed under a N_2_ atmosphere at a heating rate of 10 °C min^−1^ from 25 °C to 800 °C. Figure S3 show the TG curves of (H_3_Cyclen)(ZnBr_4_)·Br·H_2_O. The loss of free water molecules per unit cell begins at 92 °C (observed 1.62%, calculated 2.73%). After 239 °C, a rapid mass loss occurs with increasing temperature, corresponding to the decomposition of the organic cations and the collapse of the entire framework structure.

### 2.3. Optical Properties

**Figure 3:**
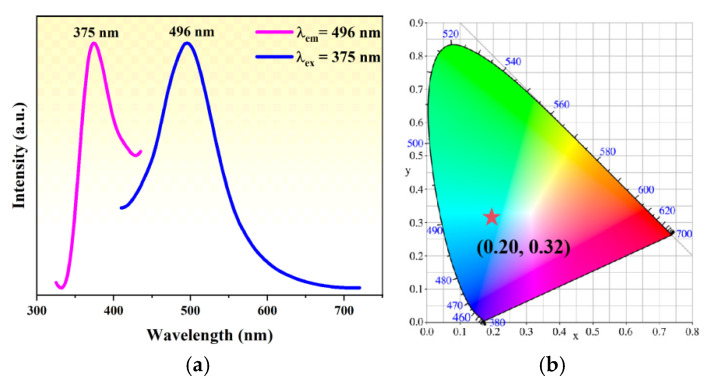
(**a**) Solid-state excitation and emission spectra. (**b**) CIE coordinates diagram of (H_3_Cyclen)(ZnBr_4_)·Br·H_2_O.

The excitation and emission spectra of (H_3_Cyclen)(ZnBr_4_)·Br·H_2_O were measured. As shown in Figure 3a, under an excitation wavelength of 375 nm, the compound exhibits a blue-green emission peak at 496 nm with a Stokes shift of 121 nm. The CIE chromaticity coordinates calculated from the emission spectrum are (0.20, 0.32) (Figure 3b). Under irradiation with a 365 nm UV lamp, (H_3_Cyclen)(ZnBr_4_)·Br·H_2_O exhibits varying degrees of blue-green emission. The photographed luminescence images (Figure S4) are in good agreement with the calculated CIE coordinates.

The time-resolved decay curve of (H_3_Cyclen)(ZnBr_4_)·Br·H_2_O was measured and fitted with a double-exponential equation (Figure S5). The fitting result shows that the fluorescence lifetime is 27.63 ns, indicating that the compound is a fluorescent material, which is comparable to the lifetimes reported for other Zn(II)-based halides [25,26,27].

To gain deeper insight into the luminescence mechanism of the compound, solid-state UV–Vis absorption spectroscopy was performed using a PE Lambda 900 spectrophotometer with BaSO_4_ as the reference standard. (H_3_Cyclen)(ZnBr_4_)·Br·H_2_O was measured in the wavelength range of 200-800 nm. The absorption spectrum and Tauc plot of (H_3_Cyclen)(ZnBr_4_)·Br·H_2_O are shown in Figure S6. From the Tauc plot, the optical band gap (Eg) was determined to be 4.94 eV [28,29,30,31].

**Figure 4:**
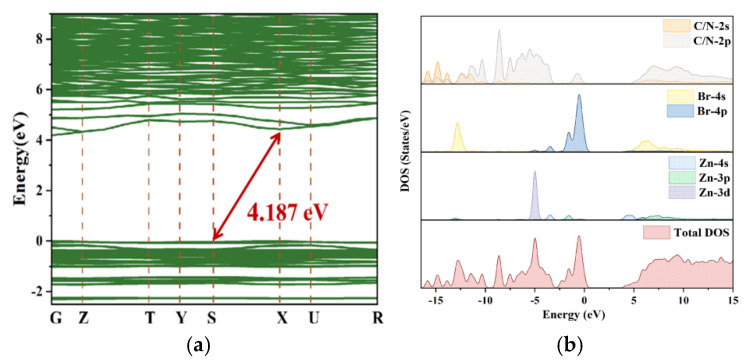
Band structure (**a**) and density of states (**b**) of (H_3_Cyclen)(ZnBr_4_)·Br·H_2_O.

To further investigate the luminescence mechanism of the compound, density functional theory (DFT) calculations were performed to determine the band gap and density of states (DOS). The results are shown in Figure 4. The calculated band gap of (H_3_Cyclen)(ZnBr_4_)·Br·H_2_O is 4.187 eV (Figure 4a). The valence band maximum (VBM) is located at the S point, while the conduction band minimum (CBM) is located at the X point, indicating an indirect band gap character. The calculated band gap is lower than the optical band gap obtained from the Tauc plot fitting, which is attributed to the inherent limitations of DFT calculations that tend to underestimate band gap values [32,33]. As revealed by the projected density of states (PDOS), the VBM are contributed by Br-4p orbital and the CBM are due to Zn-4s orbital (Figure 4b). Therefore, the luminescence of (H_3_Cyclen)(ZnBr_4_)·Br·H_2_O mainly originates from the inorganic component. The possible mechanism is as follows: upon photon absorption by the OIMH material, electrons are excited from the valence band to the conduction band, forming an exciton through Coulomb interaction. Due to the zero-dimensional structure of (H_3_Cyclen)(ZnBr_4_)·Br·H_2_O, the presence of the exciton strongly distorts the surrounding lattice, thereby “trapping” itself to form a self-trapped exciton (STE) [34,35,36,37,38], which ultimately emits blue-green fluorescence. This is also consistent with the large Stokes shift observed for (H_3_Cyclen)(ZnBr_4_)·Br·H_2_O.

**Figure 5:**
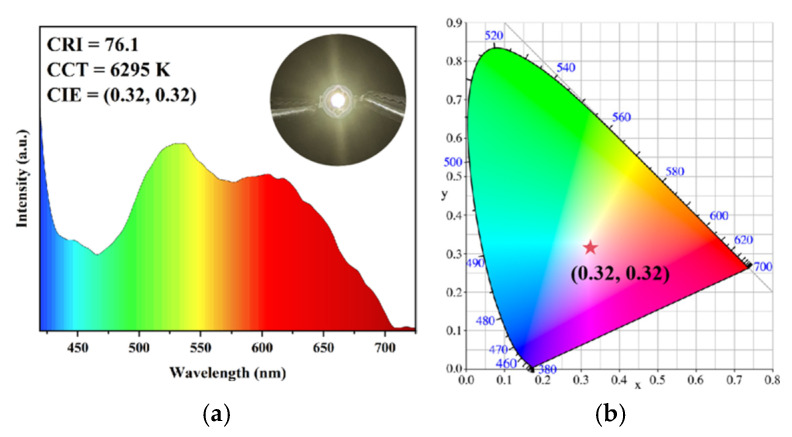
The emission spectrum (**a**) and CIE chromaticity coordinates (**b**) of the coated LED.

Since (H_3_Cyclen)(ZnBr_4_)·Br·H_2_O emits blue-green light under a 365 nm UV lamp, we successfully fabricated white light-emitting diodes (WLEDs) by uniformly mixing the as-synthesized sample powder with commercial red phosphor Y_2_O_3_:Eu^3+^ and commercial green phosphor (Ce,Tb)MgAl_11_O_19_ using epoxy resin, and then coating the mixture onto a 365 nm UV LED chip. Figure 5 shows the electroluminescence spectra (with photographs of the illuminated WLEDs in the insets) (Figure 5a) and CIE chromaticity diagrams of (H_3_Cyclen)(ZnBr_4_)·Br·H_2_O driven at a voltage of 3.5 V and a current of 20 mA (Figure 5b). The color rendering index (CRI) is 76.1, and the CIE coordinates are (0.32, 0.32), which fall within the cold white light range of LEDs. These results indicate that (H_3_Cyclen)(ZnBr_4_)·Br·H_2_O is a promising phosphor material.

## 3. Experimental Section

### 3.1. Materials and Methods

The reagents were all purchased from commercial sources and used directly without further purification. Single-crystal X-ray diffraction data for (H_3_Cyclen)(ZnBr_4_)·Br·H_2_O was collected on a Bruker D8 QUEST diffractometer equipped with Mo−Kα radiation (λ = 0.71073 Å). The crystal structures were solved and refined using the SHELXL-2018/3 program within OLEX2 [39,40]. All non-hydrogen atoms were refined with anisotropic displacement parameters, while hydrogen atoms on carbon and nitrogen atoms were generated geometrically and placed in idealized positions. Crystal data collection and refinement parameters are summarized in Table 1, and their selected bond lengths and angles are provided in Tables S1 and S2, respectively. Powder X-ray diffraction (PXRD) patterns were recorded on a Panalytical X’pert3 diffractometer with graphite-monochromated Cu/Kα radiation (λ = 0.154 nm). Infrared spectra were obtained on a Nicolet iS20 Fourier-transform infrared spectrometer with 16 scans over the wavenumber range of 4000–400 cm^−1^. Thermogravimetric analysis was performed on a Netzsch STA449F3 Jupiter thermal analyzer under N_2_ atmosphere from 25 °C to 800 °C at a heating rate of 10 °C min^−1^. UV–vis absorption spectra were measured using a PE Lambda 900 spectrophotometer in the wavelength range of 200–800 nm. Excitation and emission spectra for fluorescence properties were recorded on a Hitachi F-4600 fluorescence spectrophotometer. Time-resolved decay curves were obtained using an Edinburgh FLS-1000 fluorescence spectrometer equipped with a picosecond pulsed diode laser. White light-emitting diodes (WLEDs) were fabricated using a red phosphor Y_2_O_3_:Eu^3+^, a green phosphor (Ce,Tb)MgAl_11_O_19_, and a 365 nm chip.

### 3.2. Synthesis of (H_3_Cyclen)(ZnBr_4_)·Br·H_2_O

ZnBr_2_ (0.5 mmol, 0.113 g) and Cyclen (0.5 mmol, 0.086 g) were dissolved in a mixed solution of 1 mL hydrobromic acid and 5 mL ethanol. The resulting suspension was heated and stirred on a magnetic stirrer for 10 min, then allowed to cool to room temperature and evaporate slowly. After three days, colorless crystals precipitated. The crystals were filtered off and washed three times with methanol. The structure was determined to be (H_3_Cyclen)(ZnBr_4_)·Br·H_2_O (C_8_H_25_Br_5_N_4_OZn, M_r_ = 658.24) by single-crystal X-ray diffraction. The yield was 43%.

## 4. Conclusions

In summary, we have synthesized a novel organic–inorganic hybrid metal halide (OIMH) material, (H_3_Cyclen)(ZnBr_4_)·Br·H_2_O, using ZnBr_2_ as the inorganic component and Cyclen as the organic component. The compound was characterized by single-crystal X-ray diffraction, powder X-ray diffraction, infrared spectroscopy, and thermogravimetric analysis. Structural analysis reveals that (H_3_Cyclen)(ZnBr_4_)·Br·H_2_O comprises H_3_Cyclen^3+^ cations, [ZnBr_4_]^2−^ tetrahedra, and free Br^−^ ions and water molecules. The [ZnBr_4_]^2−^ units are uniformly separated by the H_3_Cyclen^3+^ cations, resulting in an overall 0D structure. This compound emits bright blue-green light under a UV lamp, which prompted a detailed investigation of its luminescence properties. Under an excitation wavelength of 375 nm, the compound exhibits an emission peak at 496 nm with a Stokes shift of 121 nm, and a fluorescence lifetime of 27.63 ns. UV–Vis absorption spectroscopy and DFT calculations indicate that the luminescence of (H_3_Cyclen)(ZnBr_4_)·Br·H_2_O mainly originates from self-trapped excitons. The bright blue-green emission suggests that this compound is a potential phosphor material. Therefore, (H_3_Cyclen)(ZnBr_4_)·Br·H_2_O was mixed with commercial phosphors to fabricate a WLED. The resulting WLED exhibits a CRI of 76.1 and CIE coordinates of (0.32, 0.32). We will continue to focus on luminescent OIMH materials and aim to develop OIMH materials with even better luminescent performance.

## Figures and Tables

**Table 1:** Crystallographic data for (H_3_Cyclen)(ZnBr_4_)·Br·H_2_O.

	(H_3_Cyclen)(ZnBr_4_)·Br·H_2_O
Formula	C_8_H_25_Br_5_N_4_OZn
Fw	658.24
Crystal system	Orthorhombic
Space group	*P*2_1_2_1_2_1_
a/Å	7.682(6)
b/Å	15.184(12)
c/Å	16.576(16)
α/°	90
β/°	90
γ/°	90
V/Å^3^	1933(3)
Z	4
ρ_calc_g/cm^3^	2.261
Independent reflections	4715 [R_int_ = 0.0709, R_sigma_ = 0.0751]
GOF F^2^	0.981
Final R indexes [I>2σ (I)]	R_1_ = 0.0362, wR_2_ = 0.0507
Final R indexes [all data]	R_1_ = 0.0720, wR_2_ = 0.0580

## Data Availability

The original contributions presented in this study are included in the Supplementary Material. Further inquiries can be directed to the corresponding authors.

## Supplementary Materials

The following supporting information can be downloaded at: https://www.mdpi.com/article/10.3390/molecules31122082/s1, Figure S1: PXRD patterns of (H_3_Cyclen)(ZnBr_4_)·Br·H_2_O; Figure S2: Infrared spectrum of (H_3_Cyclen)(ZnBr_4_)·Br·H_2_O; Figure S3: TG curves of (H_3_Cyclen)(ZnBr_4_)·Br·H_2_O; Figure S4: The photo images of bulk crystal of (H_3_Cyclen)(ZnBr_4_)·Br·H_2_O; Figure S5: Solid-state luminescence lifetime of (H_3_Cyclen)(ZnBr_4_)·Br·H_2_O; Figure S6: UV-vis absorption spectrum (a) and Kubelka-Munk converted diffuse reflectance spectrum (b) of (H_3_Cyclen)(ZnBr_4_)·Br·H_2_O; Table S1: Bond lengths and bond angles for (H_3_Cyclen)(ZnBr_4_)·Br·H_2_O; Table S2: Hydrogen bonds for (H_3_Cyclen)(ZnBr_4_)·Br·H_2_O.

## References

[1] Wang G.X., Meng L.C., Liu X.Y., Sun Y.W., Zhang L., Fatehi P., Xiao H.N., Wu W.B. (2026). Luminescent metal-organic frameworks for gas-phase detection: Mechanisms, methods and applications. Coord. Chem. Rev..

[2] Gu X.T., Li Y.Y., Jia Y., Li Y.Y. (2026). Unlocking the next generation: Nanoclusters as advanced electrochemiluminescence emitters for biosensing. Chem. Eng. J..

[3] Han Z.S., Huo J.T., Zhou H.C. (2026). Function Decoupling and Modular Platform: Emerging Design Principles for MOF Luminescent Sensing. Acc. Chem. Res..

[4] Perala R.S., Singh B.P., Kim M.J. (2026). Research progress in surface modification strategies for lanthanide-doped luminescent materials towards theranostic application. Coord. Chem. Rev..

[5] Chen L., Zhou C., Che J.X., Yu H., Yang M., Lv Z.J., Dong X.T. (2026). Synergistic mechanism from carbon dot modification and rare earth doping in perovskite materials for achieving tunable light color. J. Alloys Compd..

[6] Li Z.J., Li K.J., Zhao J.Q., Guo D.X., Lu R.Y., Sheng T.Q., Fu Z.L. (2026). Lattice-Regulated CaNb_2_O_6_ Niobate for High-Performance NIR-II Luminescent Thermometry and Flexible Optical Sensing. Inorg. Chem..

[7] Tan F.S., Ma H., Fan Q.M., Wan J., Tang H.J., Ren P., Zhou Q., Wang Z.L. (2026). Highly efficient Sb-doped zinc chloride hybrid for single-component white light emission and anti-counterfeiting applications. J. Mol. Struct..

[8] Sun C.N., Wang K.K., Wang F., Zhao X.X., Bai S., Su B.B., Xie H.D. (2026). Near-unity quantum yield blue hybrid cuprous halide with high water resistance for multiple applications. Chem. Eng. J..

[9] Wang D., Kang H.L., Wang Z. (2026). Research progress on organic-inorganic hybrid metal halide based long-lived luminescent materials. Coord. Chem. Rev..

[10] Chen C., Dong G.K., Qi H.B., Zhang J., Li J., Wu R.R., Wu W.Z. (2026). Sb^3+^-doped (TEA)_2_HfCl_6_ metal halide for White Light Emissionwhite light emission and Anti-Counterfeiting Encryptionanti-counterfeiting encryption. J. Alloys Compd..

[11] You M.X., Lyu Z., Liu S.Y., Peng G., Shen S.D., Tan T.X., Wei S., You H.P., Liao W.P. (2026). Organic cation engineering enabled tunable emissions in indium-based metal halides for versatile applications. Mater. Today Chem..

[12] Li H.P., Han B., Jiang Z.Y., Dou Z.D., Ma X.R., Ma X.Q., Tan Z. (2026). Near-Unity Green Luminescent Hybrid Manganese Halides: Ionothermal Synthesis and White Light-Emitting Diode Applications. Cryst. Growth Des..

[13] Garsed R., Hernan G., Perles J., Martínez J.I., Díaz A.C., Cantelar E., Zamora F., Troyano J., Amo-Ochoa P. (2026). Dual-Emission Switching in a Mn(II)-Based Hybrid Bromide via Water Coordination: Synthesis, Structure, and Processing. Adv. Opt. Mater..

[14] Zhang Q., Huang T.W., Liu Z.Y., Feng Y.N., Yu Y., Li L.Y. (2025). Hydrogen bonding evolution and efficient blue light emission in a series of Zn-based organic-inorganic hybrid metal halide crystals. Sci. China Mater..

[15] Cui Y.B., Lin J.W., Liu K.J., Shao Y.H., Zhao D., Guo Z.N., Zhao J., Xia Z.G., Liu Q.L. (2025). Tuning covalent bonding in zinc-based hybrid halides towards tunable room-temperature phosphorescence. Chem. Sci..

[16] Golovnev N.N., Gerasimova M.A., Molokeev M.S., Plyaskin M.E., Baronin M.E. (2022). Photoluminescence of pefloxacindi-ium manganese(II) and zinc(II) tetrahalides. J. Mol. Struct..

[17] Zhang J., Ma Y.X., Wu M., He Q., Chen S.Y., Ju P., He Y.C., Lei X.W. (2024). Zero-dimensional organic-inorganic hybrid zinc halide with stable broadband blue light emissions. CrystEngComm.

[18] Jin Y.L., Li J.W., Xu Z.H., Ye L.W., Wu D.W., Gao Y.G., Zhuang X.X. (2025). High-efficiency green emission and temperature-modulated dual photoluminescence in a zero-dimensional zinc bromide hybrid. J. Alloys Compd..

[19] Cong L., Jia Y.X., Cheng X.H., Liu Y., Li J., Cui B.B. (2025). Recent Advances in Low-Dimensional Organic-Inorganic Hybrid Metal Halides (0D-2D) for Solid-State Lighting. Adv. Opt. Mater..

[20] Liu Z.X., Zhang J.Y., Xu Y.F., Li P.C., Zhao X.C., Zhou W., Wang S.Y., Liu W.F. (2025). Crystal structure and optical properties characterization in quasi-0D lead-free organic-inorganic hybrid crystals (C_6_H_16_N)_2_MX_4_ (M = Zn, Mn; X = Br, Cl). J. Solid State Chem..

[21] Wu Y.J., Xu Y.K., Lei Y.T., Peng G.Q., Zhou Y.H., Li Q.J., Li Z.H., Wang Q., Jin Z.W. (2025). Zero-Dimensional Metal Halides Inorganic Frameworks Modulation for Sensitivity and Stable Direct X-Ray Detection. Laser Photonics Rev..

[22] Spackman P.R., Turner M.J., McKinnon J.J., Wolff S.K., Grimwood D.J., Jayatilaka D., Spackman M.A. (2021). CrystalExplorer: A program for Hirshfeld surface analysis, visualization and quantitative analysis of molecular crystals. J. Appl. Crystallogr..

[23] He Y.C., Ge G.Z., Li S.X., Zhang J., Liu Z.X., Bi C.Y. (2026). Boosting room-temperature phosphorescence of Lead(II)-based coordination polymers via structural rigidification with auxiliary ligands. J. Mol. Struct..

[24] Spackman M.A., Jayatilaka D. (2009). Hirshfeld surface analysis. CrystEngComm.

[25] Huang T., Wang Z.X., Li T.Z., Shen X.D., Liang W.Z., Niu Q., Zhong X.C., Zou B.S. (2024). Multifunctional Phosphor with High-Efficient Near-Infrared Emission Based on Antimony-Zinc Halides. ACS Appl. Mater. Interfaces.

[26] Zhou J.Q., Lin J.W., Guo Z., Xie P.R., Chen C.C., Mao L.L. (2024). Tunable Blue-Light-Emitting Organic-Inorganic Zinc Halides with Thermally Activated Delayed Fluorescence and Room-Temperature Phosphorescence. ACS Appl. Mater. Interfaces.

[27] Liu Y.H., Wang W.Q., Zhang B.L., Wang Y.J., Ren M.P., Jing Z.H., Yue C.Y. (2023). Zero-dimensional organic-inorganic hybrid zinc halide with broadband yellow light emission. CrystEngComm.

[28] Fattal H., Creason T.D., Delzer C.J., Yangui A., Hayward J.P., Ross B.J., Du M.H., Glatzhofer D.T., Saparov B. (2021). Zero-Dimensional Hybrid Organic-Inorganic Indium Bromide with Blue Emission. Inorg. Chem..

[29] He Y.C., Ge G.Z., Bi C.Y., Zhong Y., Zhao F.H. (2026). Three Novel Alkaline-Earth Ca(II)/Mg(II)/Ba(II)-Based Coordination Polymers as Fluorescent Probes for Nor and Pef Antibiotic Detection. Cryst. Growth Des..

[30] Wang Z.Y., Li Y.K., Sun C., Li D.Y., Peng C.D., Fei H.H. (2025). Introducing Methoxy Functionality to Modulate the Lead Halide Dimensionality in Robust Metal-Organic Frameworks for Enhanced Broadband Emission. Adv. Opt. Mater..

[31] Ren X.F., Li X.H., Li M.T., Dong Y.H., Du C., He Y.C., Jing Z.H. (2025). Syntheses, structures, Hirshfeld surface analyses and band gap analyses of five Cd(II)/Zn(II)-based coordination compounds. J. Mol. Struct..

[32] Perdew J.P., Levy M. (1983). Physical Content of the Exact Kohn-Sham Orbital Energies: Band Gaps and Derivative Discontinuities. Phys. Rev. Lett..

[33] Song X.L., Wang S.H., Yang Y., Zhou Y.L., Huang X., Tang B., Liu H.M. (2024). Halogen Content Tunes the Color Temperature of White Light and Afterglow in Zero-Dimensional Hybrid Indium Halides. Chem. Mater..

[34] You Z.L., Xiang Z.X., Wei J.H., Wang T.C., He Z.L., Chen K.L., Kuang D.B. (2026). Spatiotemporally Resolved Anti-Counterfeiting via Multicomponent Zero-Dimensional Metal Halides with Anti-Kasha Emission. Adv. Opt. Mater..

[35] Cao Q.Y., Yao B.L., Wang Y., Pang M.M., Min H., Zhu Y.Q., Shao D. (2026). Steric hindrance-driven structure engineering of hybrid Cu(I) halides for modulated optical performance. J. Mol. Struct..

[36] Dastidar R.G., Okamoto T., Takahashi K., Rana S., Awasthi K., Ohta N., Subrahmanyam C., Biju V. (2026). Photoluminescence Enhancement in Zero-Dimensional Hybrid Copper Halide Single Crystals. Chem. Mater..

[37] Zhang R.Y., Dan N., Gao X.N., Fu H.R., Guo Y.M., Ma L.F. (2025). Structural Transformation and Luminescence Switching of Hybrid Antimony Halides for Single-Component White Light and Encryption. Inorg. Chem..

[38] Wang X.C., Bai T.X., Sun J.L., Liu J.Y., Su Y., Chen J.S. (2024). The effect of solvent on the formation of low-dimensional metal halides and their self-trapped exciton emission. Chem. Eng. J..

[39] Dolomanov O., Bourhis L., Gildea R., Howard J., Puschmann H. (2009). OLEX2: A complete structure solution, refinement and analysis program. J. Appl. Crystallogr..

[40] Sheldrick G.M. (2015). Crystal structure refinement with SHELXL. Acta Crystallogr. C Struct. Chem..

