# Supplementary material for: A Zero-Dimensional Zn(II)-Based Organic–Inorganic Hybrid Metal Halide with Blue-Green Emission for White Light-Emitting Diode Application
Source: Molecules. 2026 Jun 13;31(12):2082. doi: 10.3390/molecules31122082 (PMC13305100; doi:10.3390/molecules31122082)

checkCIF/PLATON report

Structure factors have been supplied for datablock(s) 240124_c6_0m

THIS REPORT IS FOR GUIDANCE ONLY. IF USED AS PART OF A REVIEW PROCEDURE FOR PUBLICATION, IT SHOULD NOT REPLACE THE EXPERTISE OF AN EXPERIENCED CRYSTALLOGRAPHIC REFEREE.

No syntax errors found. CIF dictionary Interpreting this report

Datablock: 240124_c6_0m

```
Bond precision:   C-C = 0.0097 A          Wavelength=0.71073

Cell:               a=7.682(6)     b=15.184(12)      c=16.576(16)
                    alpha=90       beta=90           gamma=90
Temperature:        273 K

                 Calculated                    Reported
Volume           1934(3)                       1933(3)
Space group      P 21 21 21                    P 21 21 21
Hall group       P 2ac 2ab                     P 2ac 2ab
Moiety formula   C8 H23 N4, Br4 Zn, Br, H2 O   Br, Br4 Zn, C8 H23 N4, H2 O
Sum formula      C8 H25 Br5 N4 O Zn            C8 H25 Br5 N4 O Zn
Mr               658.21                        658.24
Dx,g cm-3        2.261                         2.261
Z                4                             4
Mu (mm-1)        11.598                        11.602
F000             1256.0                        1256.0
F000'            1252.48
h,k,lmax         10,20,22                      10,20,22
Nref             4771[ 2717]                   4715
Tmin,Tmax        0.271,0.313                   0.443,0.746
Tmin'            0.228

Correction method= # Reported T Limits: Tmin=0.443 Tmax=0.746
AbsCorr = MULTI-SCAN

Data completeness= 1.74/0.99        Theta(max)= 28.193

                                                      wR2(reflections)=
R(reflections)= 0.0362( 3351)                         0.0580( 4715)

S = 0.981                    Npar= 179
```

The following ALERTS were generated. Each ALERT has the format
test-name_ALERT_alert-type_alert-level.
Click on the hyperlinks for more details of the test.

Alert level C

```
PLAT042_ALERT_1_C Calc. and Reported MoietyFormula Strings  Differ     Please Check
              Calc: C8 H23 N4, Br4 Zn, Br, H2 O
              Rep.: Br, Br4 Zn, C8 H23 N4, H2 O
PLAT341_ALERT_3_C Low Bond Precision on  C-C Bonds ...............    0.00975 Ang.
```

Alert level G

```
PLAT002_ALERT_2_G Number of Distance or Angle Restraints on AtSite          2 Note
PLAT007_ALERT_5_G Number of Unrefined Donor-H Atoms ..............          8 Report
              H1A   H1B   H3A   H3B   H4A   H4B   H1E   H1F
PLAT172_ALERT_4_G The CIF-Embedded .res File Contains DFIX Records          1 Report
PLAT199_ALERT_1_G Reported _cell_measurement_temperature ..... (K)        273 Check
PLAT200_ALERT_1_G Reported   _diffrn_ambient_temperature ..... (K)        273 Check
PLAT720_ALERT_4_G Number of Unusual/Non-Standard Labels ..........          1 Note
              Zn05
PLAT790_ALERT_4_G Centre of Gravity not Within Unit Cell: Resd.  #          4 Note
              H2 O
PLAT794_ALERT_5_G Tentative Bond Valency for Zn05      (II)      .       1.92 Info
PLAT860_ALERT_3_G Number of Least-Squares Restraints .............          1 Note
PLAT912_ALERT_4_G Missing # of FCF Reflections Above STh/L=  0.600          6 Note
PLAT969_ALERT_5_G The 'Henn et al.' R-Factor-gap value ...........      1.033 Note
              Predicted wR2: Based on SigI**2  5.61 or SHELX Weight  5.91
PLAT978_ALERT_2_G Number C-C Bonds with Positive Residual Density.          0 Info
```

0 **ALERT level A** = Most likely a serious problem - resolve or explain
0 **ALERT level B** = A potentially serious problem, consider carefully
2 **ALERT level C** = Check. Ensure it is not caused by an omission or oversight
12 **ALERT level G** = General information/check it is not something unexpected

3 ALERT type 1 CIF construction/syntax error, inconsistent or missing data
2 ALERT type 2 Indicator that the structure model may be wrong or deficient
2 ALERT type 3 Indicator that the structure quality may be low
4 ALERT type 4 Improvement, methodology, query or suggestion
3 ALERT type 5 Informative message, check

It is advisable to attempt to resolve as many as possible of the alerts in all categories. Often the minor alerts point to easily fixed oversights, errors and omissions in your CIF or refinement strategy, so attention to these fine details can be worthwhile. In order to resolve some of the more serious problems it may be necessary to carry out additional measurements or structure refinements. However, the purpose of your study may justify the reported deviations and the more serious of these should normally be commented upon in the discussion or experimental section of a paper or in the "special_details" fields of the CIF. checkCIF was carefully designed to identify outliers and unusual parameters, but every test has its limitations and alerts that are not important in a particular case may appear. Conversely, the absence of alerts does not guarantee there are no aspects of the results needing attention. It is up to the individual to critically assess their own results and, if necessary, seek expert advice.

Publication of your CIF in IUCr journals

A basic structural check has been run on your CIF. These basic checks will be run on all CIFs submitted for publication in IUCr journals (*Acta Crystallographica*, *Journal of Applied Crystallography*, *Journal of Synchrotron Radiation*); however, if you intend to submit to *Acta Crystallographica Section C* or *E* or *IUCrData*, you should make sure that full publication checks are run on the final version of your CIF prior to submission.

Publication of your CIF in other journals

Please refer to the *Notes for Authors* of the relevant journal for any special instructions relating to CIF submission.

PLATON version of 02/02/2025; check.def file version of 02/02/2025

Datablock 240124_c6_0m - ellipsoid plot

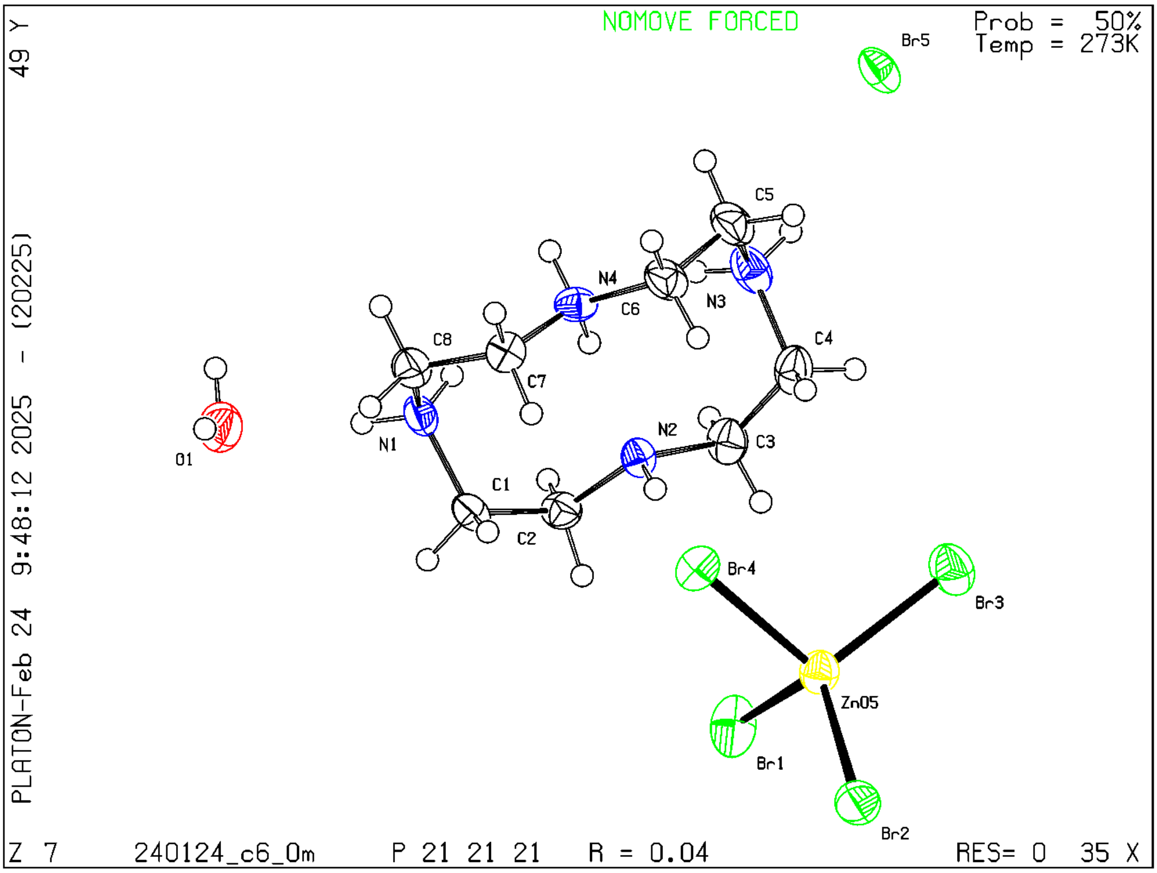

Supplement: Supplementary file 1 [file molecules-31-02082-s001.zip › 240124_C6_0m_cifreport.pdf]
