# Supplementary material for: A Zero-Dimensional Zn(II)-Based Organic–Inorganic Hybrid Metal Halide with Blue-Green Emission for White Light-Emitting Diode Application
Source: Molecules. 2026 Jun 13;31(12):2082. doi: 10.3390/molecules31122082 (PMC13305100; doi:10.3390/molecules31122082)
Supplement: Supplementary file 1 [file molecules-31-02082-s001.zip › Supporting information.pdf]

Supporting information

A Zero-Dimensional Zn(II)-Based Organic-Inorganic Hybrid Metal Halide with Blue-Green Emission for White Light-Emitting Diode Application

Hua-Peng Liu[1,*], Yu-Chen Wang[2], Zhen-Chao Hu[1] and Yuan-Chun He[2,*]

[1] Key Laboratory for Medical Functional Nanomaterials, College of Medical Engineering, Jining Medical University, Jining, 272067, P. R. China

[2] Key Laboratory of Catalytic Conversion and Clean Energy in Universities of Shandong Province, School of Chemistry and Chemical Engineering, Qufu Normal University, Qufu 273165, P. R. China

[*]Correspondence: liuhuapeng2021@mail.jnmc.edu.cn (H.-P.Liu); heyc240@nenu.edu.cn (Y.-C. He)

Theoretical calculation. The Hirshfeld surface and the corresponding two-dimensional fingerprint plots were calculated and analyzed using CrystalExplorer software. The theoretical calculation for the title compound can use the total energy code of Cambridge Sequential Total Energy Package (CASTEP) software based on density function theory. Norm conserving pseudopotential could be used for describing the relationship between ionic cores and electrons. Hence, valence electrons adopt C 2s2p, N 2s2p, Zn 3d4s, and Br 4s4p. Finally, the CASTEP code provides default values of essential convergence criteria and calculation parameters.

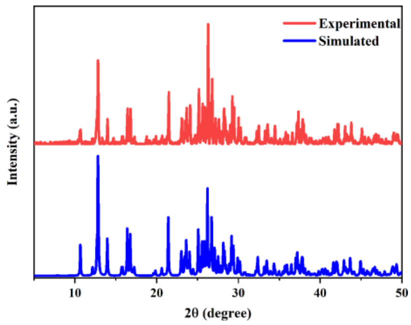

Figure S1. PXRD patterns of $(H_3Cyclen)(ZnBr_4)\cdot Br\cdot H_2O$.

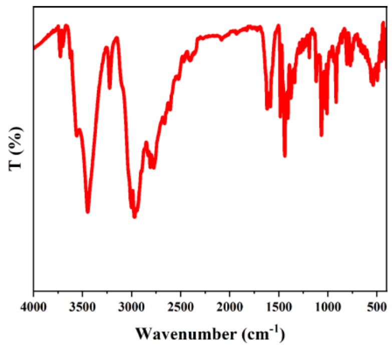

Figure S2. Infrared spectrum of $(H_3Cyclen)(ZnBr_4)\cdot Br\cdot H_2O$.

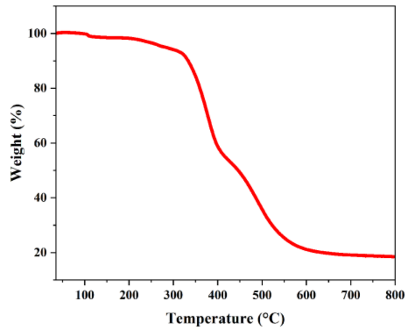

Figure S3. TG curves of $(H_3Cyclen)(ZnBr_4)\cdot Br\cdot H_2O$.

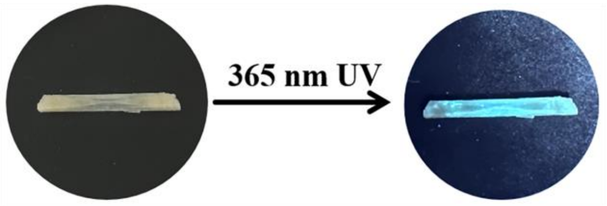

Figure S4. The photo images of bulk crystal of $(H_3Cyclen)(ZnBr_4)\cdot Br\cdot H_2O$.

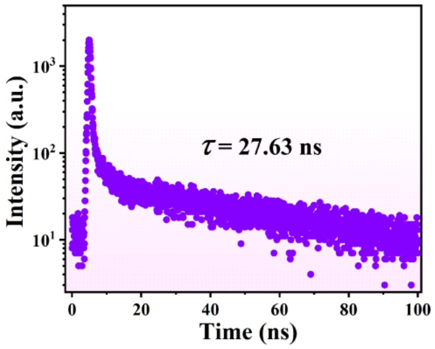

Figure S5. Solid-state luminescence lifetime of $(H_3Cyclen)(ZnBr_4)\cdot Br\cdot H_2O$.

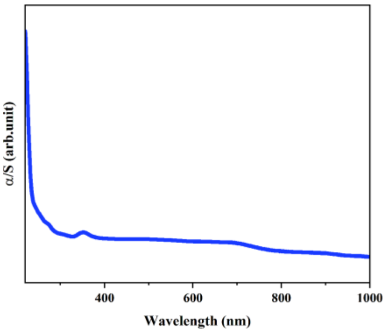

(a)

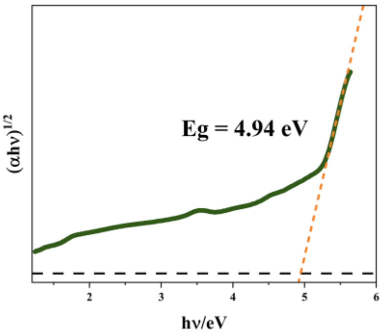

(b)

Figure S6. UV-vis absorption spectrum (**a**) and Kubelka-Munk converted diffuse reflectance spectrum (**b**) of $(H_3Cyclen)(ZnBr_4)\cdot Br\cdot H_2O$.

Table S1. Bond lengths and bond angles for $(H_3Cyclen)(ZnBr_4)\cdot Br\cdot H_2O$.

**Bond**	**Dist.**	**Bond**	**Dist.**
Br4−Zn05	2.490(3)	Br3−Zn05	2.392(2)
Br2−Zn05	2.3894(17)	Zn05−Br1	2.422(2)
**Angle**	**(°)**	**Angle**	**(°)**
Br2−Zn05−Br4	108.92(4)	Br3−Zn05−Br4	105.97(4)
Br2−Zn05−Br3	114.42(7)	Br3−Zn05−Br1	113.26(5)
Br2−Zn05−Br1	111.42(6)	Br1−Zn05−Br4	101.82(5)

Table S2. Hydrogen bonds for $(H_3Cyclen)(ZnBr_4)\cdot Br\cdot H_2O$.

D−H···A	d(D−H)	d(H···A)	d(D···A)	∠(DHA)
N4−H4B···N2	0.89	1.88	2.700(8)	151.6
N1−H1B···O1	0.89	1.98	2.810(8)	155.1
N3−H3A···Br5	0.89	2.57	3.342(6)	146.0
